# Hemodynamic benefits of celiac artery release for ruptured right gastric artery aneurysm associated with median arcuate ligament syndrome: a case report

**DOI:** 10.1186/s12893-017-0320-0

**Published:** 2017-11-28

**Authors:** Tetsuro Toriumi, Takuro Shirasu, Atsushi Akai, Yuichi Ohashi, Takatoshi Furuya, Yukihiro Nomura

**Affiliations:** 1 Department of Surgery, Asahi General Hospital, 1326, I, Asahi-shi, Chiba, 289-2511 Japan; 2 Division of Vascular Surgery, Department of Surgery, The University of Tokyo, 7-3-1, Hongo, Bunkyo-ku, Tokyo, 113-8655 Japan

**Keywords:** Median arcuate ligament syndrome, Right gastric artery aneurysm, Pancreaticoduodenal artery aneurysm, Shear stress

## Abstract

**Background:**

It has been reported that median arcuate ligament syndrome is closely associated with gastric or pancreaticoduodenal artery aneurysms. Hemodynamic state plays an important role in the formation of the aneurysms. These aneurysms are treated with open resection or endovascular exclusion. However, whether revascularization of the celiac artery can prevent the aneurysm formation is unknown. This report indicated a possibility that prophylactic revascularization for celiac artery stenosis resulted in decreased shear stress on the collaterals, which may otherwise be susceptible to new aneurysms.

**Case presentation:**

This report describes a 51-year-old man who presented with epigastric pain at our hospital. According to contrast enhanced computed tomography (CT), he was diagnosed with a ruptured right gastric artery aneurysm and celiac artery stenosis caused by the median arcuate ligament (MAL). He had a vascular anomaly of the common hepatic artery arising from the superior mesenteric artery (SMA). His vital signs were stable. We informed him of the situation and he chose open surgery rather than endovascular treatment. Following, we resected the aneurysm and transected the MAL. Intraoperative angiography after transection of the MAL showed the antegrade blood flow to the splenic artery instead of the retrograde flow via the prominent collaterals. Follow-up CT confirmed narrowed collateral vessels between the SMA and the celiac artery without de-novo aneurysms.

**Conclusion:**

While the necessity of celiac artery release could be questioned, the present case supports the hemodynamic benefits of MAL transection in terms of de-novo aneurysm prevention.

## Background

A gastric artery aneurysm is rare in visceral artery aneurysms [1, 2]. A pancreaticoduodenal artery aneurysm is also rare and sometimes associated with a compressed celiac artery caused by the median arcuate ligament (MAL). The hemodynamic state plays an important role in their development [3]. These aneurysms are treated with open resection or endovascular exclusion. Treatment depends on whether an anatomical anomaly exists, but in some cases concomitant revascularization of the celiac artery is not necessary because organ ischemia, liver ischemia for example, is unlikely. However, whether or not prophylactic celiac revascularization can prevent de-novo aneurysms remains a controversial topic due to the lack of knowledge about the hemodynamic changes after the procedure. The present report describes a case of a ruptured right gastric artery (RGA) aneurysm associated with celiac compression caused by the MAL. In this patient, though follow up time is lacking to make an appropriate conclusion, prophylactic MAL transection might have had an effect in the prevention of new aneurysm formation, in view of the hemodynamic and morphological changes in his angiography.

## Case presentation

**Fig. 1:**
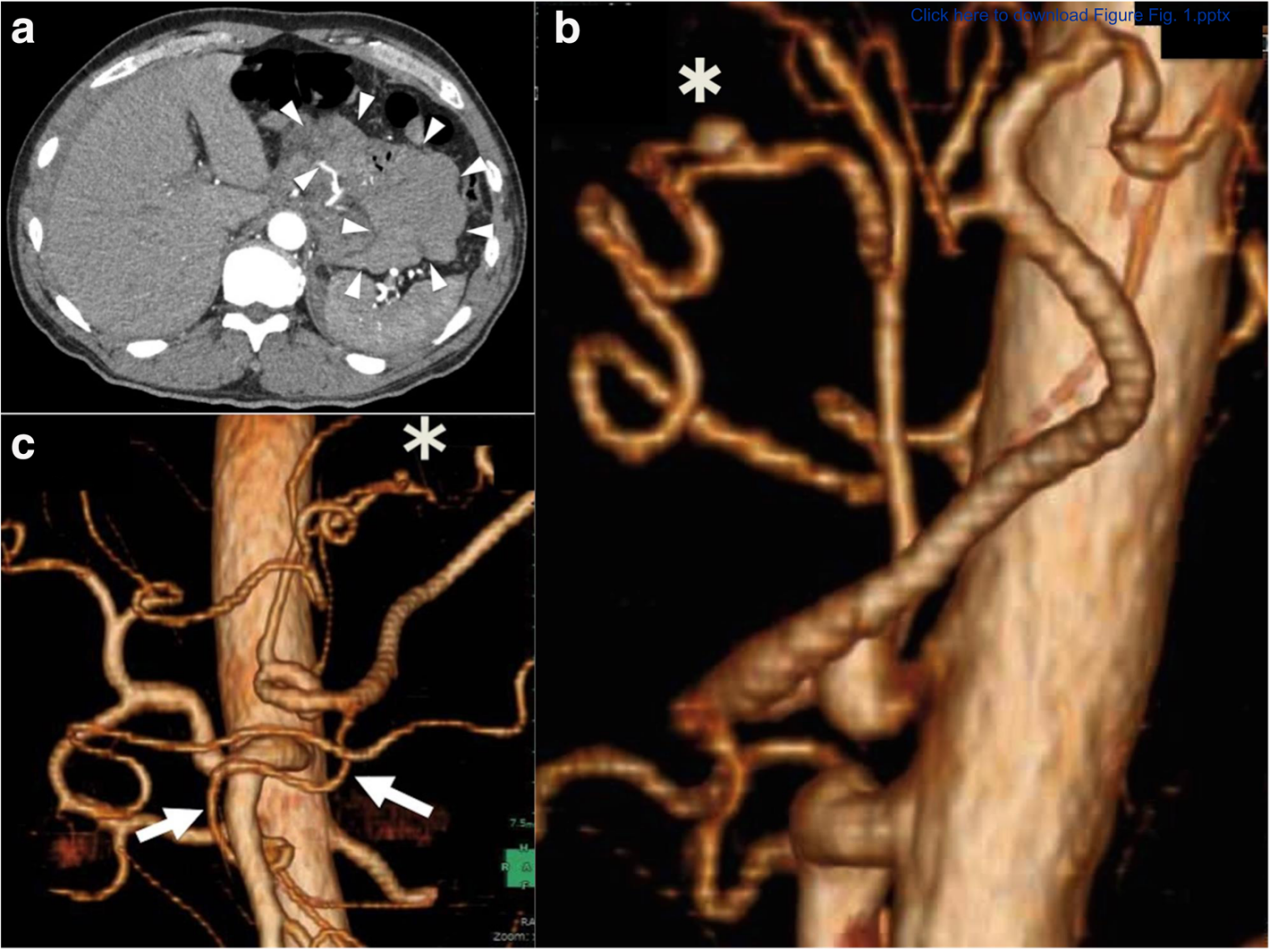
**a.** Enhanced contrast computed tomography (CT) shows a massive hematoma in the lesser omentum. **b.** In the lateral view of the aorta, the celiac axis is stenosed, most likely due to the median arcuate ligament (MAL). There is a right gastric artery aneurysm (*). **c.** 3D volume rendered CT shows a right gastric artery aneurysm (*) and an anomaly of the artery; the common hepatic artery branches from the superior mesenteric artery (SMA); the dorsal pancreatic artery (DPA) connects the SMA and the splenic artery (arrows)

A 51-year-old man with no significant medical history was admitted to our hospital with sudden onset epigastric pain. He did not take any regular medications. He had smoked a packet of cigarettes a day for 30 years and was a social drinker. On admission, his vital signs were within normal limits: body temperature of 37.0 °C, blood pressure of 113/65 mmHg, heart rate of 54 beats per minute, and oxygen saturation of 100% in room air. He complained of slight epigastric pain but there was no rebound tenderness. His hemoglobin level was 12.0 g/dL. Computed tomography (CT) with contrast enhancement showed a massive hematoma in the lesser omentum (Fig. 1a). Moreover, the root of the celiac artery was severely stenosed (3.5 mm in diameter) and a RGA aneurysm was present (Fig. 1b). A RGA aneurysm and an anomaly of the artery; common hepatic artery branches from the superior mesenteric artery (SMA) were also detected (Fig. 1c). The dorsal pancreatic artery (DPA) was dilated and clearly connecting the SMA and the splenic artery (Fig. 1c). His diagnosis was determined as median arcuate ligament syndrome (MALS) with a ruptured RGA aneurysm.

**Fig. 2:**
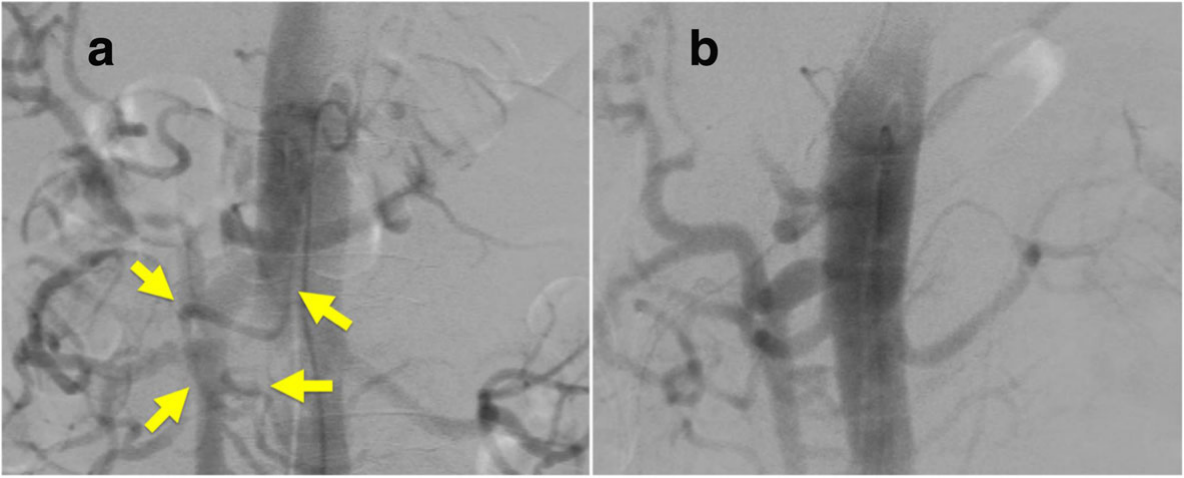
**a.** Before transection of the MAL, the DPA clearly connects the SMA and the splenic artery in the intraoperative aortogram (arrows). **b.** After transection of the MAL, the DPA is obscured, and the splenic artery is perfused by the antegrade flow from the celiac artery

**Fig. 3:**
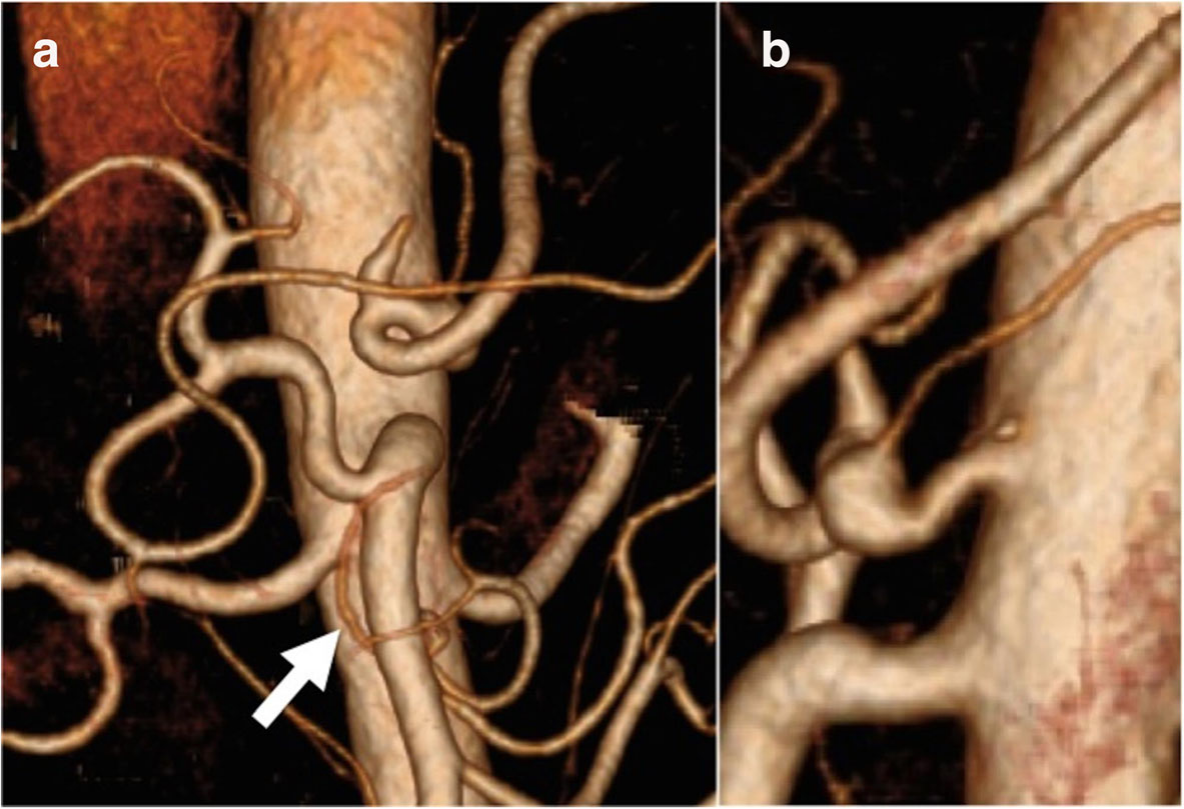
**a.** Eighteen months after surgery, the DPA is obscured (arrow). **b.** The celiac axis is enlarged and there are no new aneurysm formations

There were two treatment options, including endovascular treatment and open surgery. Endovascular treatment is less invasive. However, there is a risk of recanalization in endovascular treatment. On the other hand, we thought there were also some merits in performing open surgery. A RGA aneurysm can be treated easily by resecting the lesser omentum, and in the same operation, the MAL could also be transected. This may prevent the recurrence of the aneurysm in the future, though evidence is lacking. We informed him of the two treatment options, endovascular exclusion and open resection of the ruptured aneurysm; he chose open surgery. During emergency laparotomy, the lesser omentum with the RGA aneurysm was resected together with transection of the MAL. The intraoperative aortogram before transection of the MAL depicted the blood flow of the splenic artery coming from the SMA via the DPA (Fig. 2a). After transection of the MAL, the blood flowed from the celiac artery to the splenic artery instead of the DPA (Fig. 2b). His postoperative course was uneventful and he was discharged on postoperative day 9. Eighteen months after the operation, 3D volume rendered CT showed further enlargement of the celiac artery (5.0 mm in diameter), an obscured DPA, and no de-novo aneurysm formations (Fig. 3a, b). Informed consent was obtained from the patient prior to publication of this case report. And the CARE guidelines have been followed in this case report.

## Discussion and conclusions

MALS was first described in 1963 as a syndrome attributed to compression of the celiac artery by the MAL [4]. Although typical symptoms include epigastralgia, anorexia, and weight loss, most patients with a stenosed celiac artery caused by the MAL are asymptomatic. More recently, MALS (or compression of the celiac artery by the MAL) has attracted interest in relation to pancreaticoduodenal artery (PDA) aneurysms. Some authors have described the hemodynamic mechanisms of aneurysmal formation in the collateral vessels between the celiac artery and the SMA; that is, in the presence of compression in the celiac artery, the shear stress on the collateral vessel walls increases remarkably [5]. Once a PDA aneurysm is diagnosed, it should be treated promptly as ruptured aneurysms are life-threatening and the risk of rupture is not related to the size of the aneurysm [3].

The present report describes a case of a ruptured RGA aneurysm with MALS. The formation of an aneurysm in the gastric artery is rare [1]. Although the etiology is unclear, the main causes of gastric aneurysm are said to be atherosclerosis, trauma, and inflammation [6]. The present case did not display any of those causes; instead, we suppose the MAL played an important role in the development of this rare RGA aneurysm. In this case, the formation of a RGA aneurysm can be attributed to the increased stress on the vessel walls. Thus, the management of this patient with a ruptured RGA aneurysm and MALS should be the same as that for any patient with a PDA aneurysm and MALS. There are two proposed alternatives for the treatment of PDA aneurysms (embolization or resection), but the necessity for treatment of the stenosed celiac artery remains uncertain. Some authors have reported that celiac artery reconstruction might not be necessary given that no patients have developed organ ischemia or relapse of the aneurysm after coil embolization alone [7, 8]. However, the follow-up periods in these reports were not long enough to draw a firm conclusion, as was acknowledged by the authors. On the other hand, there are some authors who do think that revascularization or decompression of the celiac axis is necessary to prevent the possibility of recurrence of the aneurysms [9, 10]. Takase et al. reported that the follow-up period should be more than 10 years to allow the formation of new aneurysms if they are to occur; this is because they found that there was an approximately a 10-year difference in age between MALS patients with aneurysms and those without aneurysms [10]. Other reports showed that simple reconstruction of the celiac trunk led to complete aneurysm regression [11–13]. Furthermore, Mano et al. reported increasing stress on the vessel walls of the pancreaticoduodenal arcade in patients with PDA aneurysms with celiac artery occlusion [5]. These reports support the hypothesis that the MAL can independently cause aneurysm formation. They also emphasize the importance of revascularization of the celiac axis. Normalization of celiac blood flow can treat the aneurysm and prevent the formation of new aneurysms. In fact, an intraoperative aortogram before MAL transection revealed blood flow coming from the SMA via the common hepatic artery to the RGA aneurysm. Similarly, a dilated DPA was the major collateral vessel feeding the splenic artery, instead of antegrade flow through the celiac artery. If we simply resected or excluded the RGA aneurysm, the shear stress on collateral vessels such as the DPA would increase, possibly forming new aneurysms in the long-term. The findings on follow-up CT 18 months after surgery showed an enlarged celiac artery (from 3.5 mm to 5 mm in diameter) and narrowed DPA without any new aneurysm development, supporting our speculation that normalization of the celiac blood flow decreased shear stress on the collateral vessels. Nevertheless, we declare the limitation in this case report that there were no objective findings such as direct blood pressure before and after the celiac revascularization, and ideally in the long term to verify the hemodynamic evidence of our discussion. Further follow up is necessary to determine the possible recurrence of aneurysms in this patient.

In conclusion, the present report described a rare case of a ruptured RGA aneurysm in a patient with MALS. Blood flow to the common hepatic artery from the SMA might be a causative factor for this exceptional aneurysm formation in the RGA as a result of the changed hemodynamic environment. While the necessity of the celiac artery revascularization could be questioned, the present case suggested the potential benefit of MAL transection to decrease the shear stress on the collateral vessels, which are otherwise prone to the formation of new aneurysms.

## Abbreviations

CT: Computed tomography
DPA: Dorsal pancreatic artery
MAL: Median arcuate ligament
MALS: Median arcuate ligament syndrome
PDA: Pancreaticoduodenal artery
RGA: Right gastric artery
SMA: Superior mesenteric artery
